# The Potential Benefits of the Influenza Vaccination on COVID-19 Mortality Rate—A Retrospective Analysis of Patients in Poland

**DOI:** 10.3390/vaccines10010005

**Published:** 2021-12-21

**Authors:** Kinga Izabela Stańczak-Mrozek, Adam Sobczak, Leszek Lipiński, Elżbieta Sienkiewicz, Dorota Makarewicz, Roman Topór-Mądry, Jarosław Pinkas, Radosław Adam Sierpiński

**Affiliations:** 1 Medical Research Agency, Stanislawa Moniuszki 1a St., 00-014 Warsaw, Poland; kinga.stanczak-mrozek@abm.gov.pl (K.I.S.-M.); adam.sobczak@abm.gov.pl (A.S.); leszek.lipinski@abm.gov.pl (L.L.); dorota.makarewicz@abm.gov.pl (D.M.); 2 Faculty of Mathematics and Information Science, Warsaw University of Technology, Pl. Politechniki 1 St., 00-661 Warsaw, Poland; e.sienkiewicz@mini.pw.edu.pl; 3 The Agency for Health Technology Assessment and Tariff System, Przeskok 2 St., 00-032 Warsaw, Poland; sekretariat@aotm.gov.pl; 4 School of Public Health, Centre of Postgraduate Medical Education, 01-826 Warsaw, Poland; jaroslaw.pinkas@cmkp.edu.pl

**Keywords:** COVID-19, influenza vaccine, prevention, cross-protection

## Abstract

In this study, we used publicly available data from the Centrum e-Zdrowia (CeZ) Polish Databank proposing a possible correlation between influenza vaccination and mortality due to COVID-19. We limited our search to the patients with positive COVID-19 laboratory tests from 1 January 2020 to 31 March 2021 and who filled a prescription for any influenza vaccine during the 2019–2020 influenza season. In total, we included 116,277 patients and used a generalized linear model to analyze the data. We found out that patients aged 60+ who received an influenza vaccination have a lower probability of death caused by COVID-19 in comparison to unvaccinated, and the magnitude of this difference grows with age. For people below 60 years old, we did not observe an influence of the vaccination. Our results suggest a potential protective effect of the influenza vaccine on COVID-19 mortality of the elderly. Administration of the influenza vaccine before the influenza season would reduce the burden of increased influenza incidence, the risk of influenza and COVID-19 coinfection and render the essential medical resources accessible to cope with another wave of COVID-19. To our knowledge, this is the first study showing a correlation between influenza vaccination and the COVID-19 mortality rate in Poland.

## 1. Introduction

The coronavirus disease (COVID-19) is the disease caused by the SARS-CoV-2 virus. The spread of COVID-19 is now in a global pandemic phase with serious morbidity and mortality [1], causing tremendous burden on the global community. As of September 2021, more than 222 million confirmed cases of COVID-19 and over 4.5 million deaths have been reported globally [2]. Interestingly, the clinical spectrum of the illness is broad, with the severity of disease ranging from mild symptoms to severe respiratory distress syndrome [3]. Moreover, the risk factors like age greater than 60 and comorbid diseases are associated with severe outcomes and a greater risk of mortality with infection [1,4]. To date, with no highly specific antiviral drugs for SARS-CoV-2 to decrease the severity of the disease, the likelihood of hospitalization and death have been proven; however, effective vaccines against COVID-19 are in use.

Seasonal influenza occurs from fall to spring annually, and it is a major cause of worldwide morbidity, as well as mortality—Iuliano et al. estimated that up to 650,000 deaths are associated with seasonal influenza respiratory infections annually [5]. The clinical spectrum of illness caused by influenza is similar to COVID-19, including fever, chills, muscle or body aches, cough, headaches and fatigue. However, it has been reported that COVID-19 deaths occur 9.5–44.1 times more often compared to those estimated during the peak week of influenza deaths in the last seven influenza seasons in the United States [6]. As a result of the seasonality of influenza outbreaks and the continuous prevalence of COVID-19, both viruses could circulate in parallel, which elevate the potential risk of coinfection, make it difficult to distinguish between them and bring an extra burden to healthcare services. Therefore, an annual influenza vaccination is of critical importance and has long been recommended by the WHO, especially for high-risk populations, such as adults aged >65 years and pregnant women [7]. It could also have beneficial effects by reducing the risk of hospitalization due to influenza, thereby allowing healthcare workers to devote their time to patients with COVID-19 and other diseases.

In spite of the fact that SARS-CoV-2 does not mutate as often as the influenza virus, they both contain enveloped RNA as their genetic material, share similarities in structure and both contain sialic acid residues linked to glycoproteins or gangliosides as receptors for their binding proteins [8]. Moreover, numerous studies have shown that the humoral and cellular immune responses in patients coinfected with COVID-19 and influenza, as well as several of the clinical and laboratory findings, are similar in both infections [9,10,11]. Therefore, scientists have begun to look for a relationship between SARS-CoV-2 infection and influenza immunity.

Recently, several studies have suggested that prior vaccination to tuberculosis or influenza may confer a protective effect against COVID-19 [12,13,14]. Yang et al. reported a 2.44 greater odds ratio (OR) for hospitalization and 3.29 greater OR for intensive care unit admission in COVID-19-positive patients who had not received the influenza vaccination compared to the patients who were up to date on their influenza immunization [14]. The potential protective effect of influenza vaccination against COVID-19 infection has also been shown by Fink et al. They retrospectively analyzed over 92,000 COVID-19 patients from Brazil and reported 17% reduced odds of mortality, 8% lower odds of need for intensive care treatment and 18% lower odds of invasive respiratory support in patients who received a recent influenza vaccine [15]. It was also found that the odds of testing positive for COVID-19 were lower in patients vaccinated against influenza compared to those who were not (OR 0.76). Furthermore, vaccinated patients less often required hospitalization or mechanical ventilation and had a shorter hospital length of stay (OR 0.58, 0.45 and 0.76, respectively) [16]. Another study examined the role of influenza vaccine in 715,164 members of a health maintenance organization. The outcomes of this study pointed to a significant protective impact of the influenza vaccine against COVID-19 infections. Green et al. found that COVID-19 positivity was lower among individuals vaccinated for influenza during two consecutive influenza seasons (OR 0.76) in comparison to nonvaccinated individuals [17]. Wang et al. conducted a meta-analysis that systematically evaluated the association between influenza vaccination and SARS-CoV-2 infection and clinical outcomes. The analysis included 12 observational studies of 208,132 people (72,820 vaccinated and 135,112 unvaccinated). They observed a significant association between influenza vaccination and SARS-CoV-2 infection (pooled adjusted OR: 0.86). However, this study did not present any evidence of a relationship between influenza vaccination and any of the clinical outcomes (for instance, hospitalization or mortality) in COVID-19-positive patients [18].

Recent studies have also indicated that the influenza vaccine may influence both the risk of COVID-19 infection and incidence of COVID-19-related death in adults 65 years and above [19,20]. The elderly are known to be at higher risk of severe complications from COVID-19 or influenza [21,22]. Zanettini et al. reported that a 10% increase in influenza vaccination coverage was related to a statistically significant 28% decrease in the COVID-19 death rate in the elderly [19]. According to a study performed by Amato et al. influenza vaccination in adults aged 65 and over is negatively associated with COVID-19 mortality and severe clinical outcomes of COVID-19 [23]. These findings are in agreement with the results reported by Cocco et al., who indicated a possible beneficial impact of the vaccination against seasonal influenza on the incidence and severity of the SARS-CoV-2 virus in subjects aged 65 years or older [20].

On the contrary, the studies performed by Martínez-Baz et al. as well as Kissling et al., did not confirm that the influenza vaccination significantly modified the risk of SARS-CoV-2 infection, indicating an absence of an effect or a small protective effect of an influenza vaccination status on COVID-19 infections [24,25]. Furthermore, Wehenkel reported a positive association between COVID-19 deaths and the influenza vaccination of people ≥ 65 years old [26].

Despite major public health efforts on promoting influenza vaccination and the availability of various influenza vaccines in most countries, there is still a lot of skepticism regarding the influence of the influenza vaccine on outcomes of COVID-19 infections. With the influenza season upon us and the fear of a consecutive COVID-19 epidemic wave, it seems rational to further explore the relationship between influenza vaccination and COVID-19 outcomes, including mortality. Thus, in this study, we evaluated the association between influenza vaccinations administered during the 2019–2020 influenza season and mortality due to COVID-19 in Poland.

## 2. Materials and Methods

All the data for this project was obtained from a Polish databank—Centrum e-Zdrowia (CeZ)—containing anonymized information from the electronic health records of patients in Poland. Utilization of the databank for research is not considered human subjects research; therefore, the review by an institutional ethics committee was not required to conduct this study. We limited our search to include only the patients with COVID-19 laboratory test results in the CeZ from 1 January 2020 to 31 March 2021 and the patients who filled out a prescription for any influenza vaccine during the 2019–2020 influenza season.

The data relevant to COVID-19 and the influenza vaccine were extracted as follows: personal ID number, sex, date of birth and death due to COVID-19. The information on COVID-19 positivity, as well as deaths associated with COVID-19, was matched with the influenza vaccine status using the patients’ unique personal ID numbers. In total, 116,277 patients were included in our analysis. The generalized linear model was used to analyze the data, with death due to COVID-19 as a binomial response variable. All statistical analyses were performed using open software R version 4.0.2. (R Core Team (2019). R: A language and environment for statistical computing. R Foundation for Statistical Computing, Vienna, Austria. We used *p* ≤ 0.05 as the level of statistical significance.

## 3. Results

**Figure 1:**
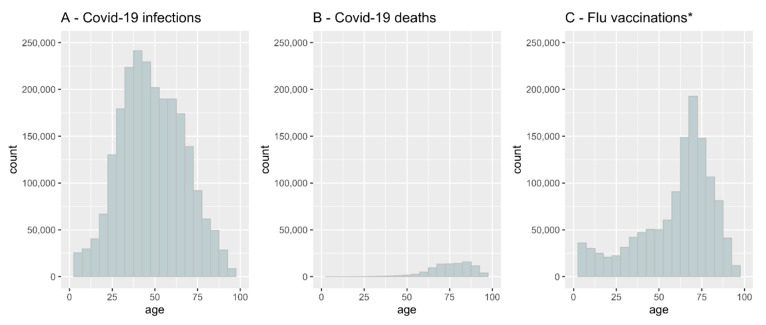
COVID-19 infection rate, COVID-19 mortality and influenza vaccination coverage by age in Poland. (**A**)- COVID-19 infection rate, (**B**)- COVID-19 mortality, (**C**)- influenza vaccination coverage. * We assumed that all the people who filled a prescription for any influenza vaccine were immunized. See the Discussion section for details.

**Figure 2:**
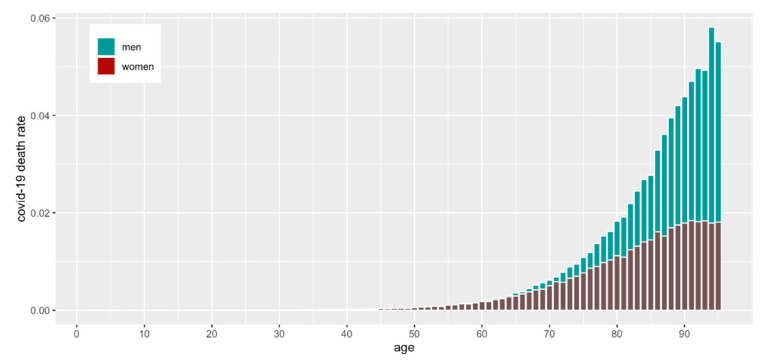
COVID-19 mortality as a percentage of the population by age and sex.

From 1 January 2020 to 31 March 2021, 2,313,496 people were determined to be COVID-19-positive in Poland (Table 1). The median age of COVID-19-infected patients was 47 years. The analysis showed that COVID-19 incidence was highest in persons aged 37 (Figure 1A). Some patients (93,208) infected with COVID-19 died (Table 1), of which 49.8 percent were male. The COVID-19 mortality rate was the highest among those aged over 62 (Figure 1B), but as a percentage of the population, it was highest among elderly males (Figure 2).

All data for influenza vaccine coverage in the total population in Poland during 2019–2020 reached 3.3%. According to the CeZ database, 1,242,204 individuals filled out a prescription for any influenza vaccine during the 2019–2020 influenza season. Some of those (9.36%) vaccinated against influenza became infected with COVID-19. There was a strong positive correlation between influenza vaccine coverage and the age of patients (Figure 1C). The highest vaccine uptake was found in patients aged 62–80 years, with a peak at 75 years. As shown in Figure 1, COVID-19-related deaths and up-take of the influenza vaccination were correlated with age.

**Figure 3:**
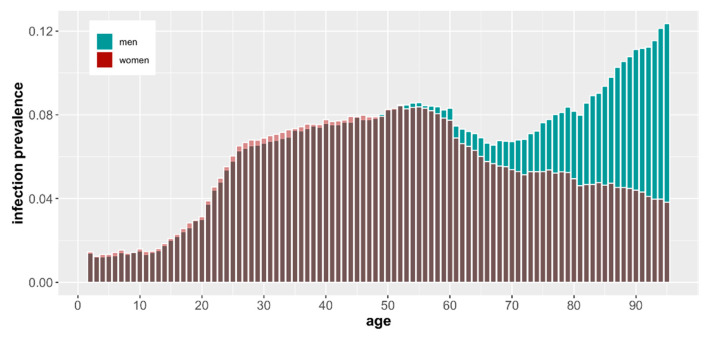
COVID-19 infection rate as a percentage of the population by age and sex based on demographic data from December 2019.

In Figure 1, we present the absolute numbers of vaccinated and infected, but it is also noteworthy to assess the numbers relative to the population size. In Figure 2 and Figure 3, we show the number of COVID-19 infections and deaths as a fraction of the population size of a given age and sex as reported in December 2019 just before the start of the pandemic by GUS (“Główny Urząd Statystyczny”, the governmental statistical body in Poland). It is clear that COVID-19 infections and deaths disproportionally affect older men (Figure 2).

**Figure 4:**
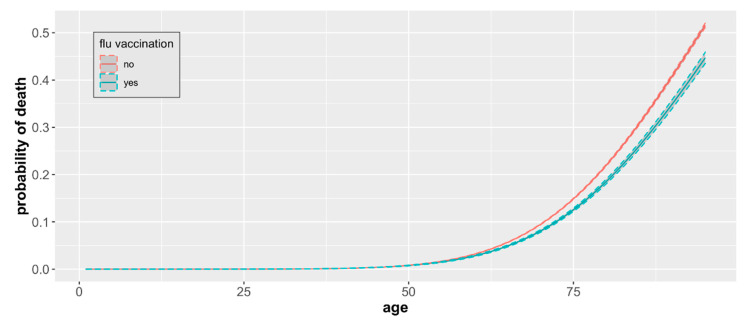
COVID-19 mortality by age and vaccination status. The probability of death from COVID-19 by age and influenza vaccination status based on a sample of 93,208 cases with observed mortality outcomes with 95% CIs.

Our analysis indicated that the impact of the influenza vaccination in the two years preceding COVID-19 infection depended on the age of the individual. For people below 60 years old, after controlling for age, there was no evidence in the data of the vaccination influence (Figure 4). The same analysis showed that there was no statistically significant association between the mortality and sex (*p* = 0.74).

**Figure 5:**
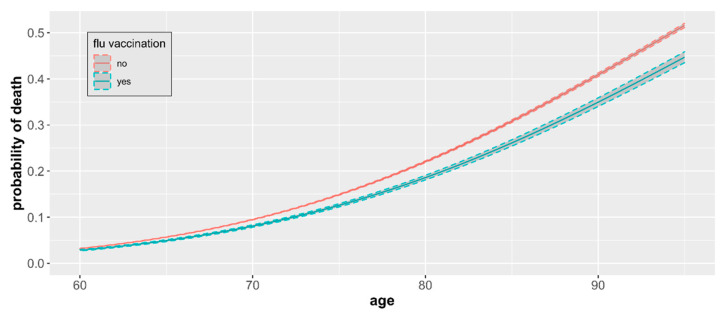
COVID-19 mortality by age and vaccination status. Estimates include only individuals 60+ years of age and represent differences in age group-specific mortality with 95% CIs.

To measure the impact of the influenza vaccine on COVID-19 mortality, a generalized linear model with a binary response was applied. Such a model can be symbolically written as: y|X ~ Bernoulli(π), with g(π) = Xβ + ε. Here, y is a binary response corresponding to the death/survival status of people infected with COVID-19, and X is a matrix with columns representing covariates, i.e., age, sex, flu vaccination status and interactions between these factors. Errors ε are iid, and the link function g(.), selected according to the Akaike’s Information Criterion [27], is defined as Φ^−1^ (inverse Gaussian distribution). A probability of death π is estimated directly from the model, with confidence intervals (Figure 4 and Figure 5) established for the entire regression curve using the Working–Hotelling method [28]. The odds ratios and their confidence intervals were calculated using the Delta method [29].

For patients 60+ years of age, we found that the influenza vaccination is associated with a lower probability of death caused by the SARS-CoV-2 virus compared to unvaccinated, and this difference, although small, remained significant (Figure 5). The magnitude of the difference increased with age.

The mean probability of death related to COVID-19 infection ranged from 0.031 among individuals aged 60 to 0.516 among individuals aged 95 in the nonvaccinated group. Mortality was consistently lower among influenza vaccinated patients across all age groups in the 60+ category and ranged from 0.028 among individuals aged 60 to 0.447 among individuals aged 95. The odds ratio of death for unvaccinated versus vaccinated individuals increased with the patient’s age and was the highest in patients aged 95 (OR 1.31, Table 2), with statistical significance (*p* < 0.05) for all age groups between 60 and 95 years old. On average, the odds of dying due to COVID-19 infection are 18% higher in the unvaccinated elderly in comparison to those vaccinated.

## 4. Discussion

Our study found no evidence for a correlation between influenza vaccination status and the risk of COVID-19-related death after a SARS-CoV-2-positive test result in the general population under 60 years of age when controlling for age. This result is in agreement with Italian study by Ragni et al. that analyzed 17,600 inhabitants from Italy and reported that influenza vaccination coverage did not affect either hospitalization or death in COVID-19 patients [30]. Another study from the U.S showed no impact of influenza vaccination on the risk of hospitalization or in-hospital mortality [31]. Our results also confirmed the previous findings of Pedote et al. and Taghioff et al., who demonstrated that the history of influenza vaccination is not associated with SARS-CoV-2 death [21,22]. There has been recent speculation, especially in social media, on the potential harmful effects of influenza vaccination in SARS-CoV-2-positive patients, but we found no evidence of a harmful effect in this retrospective study.

In the elderly cohort, we found evidence that influenza vaccination in COVID-19 patients reduces the risk of death in those aged ≥60. Our analysis showed that mortality due to COVID-19 infection in the elderly was lower among influenza-vaccinated patients compared to the unvaccinated subjects. If a causal link could be established in experimental studies, this finding would suggest that the influenza vaccination could play an important role in lowering deaths from SARS-CoV-2 infection in this particular population. It is also noteworthy that the vaccine’s protective effect seems to increase with age.

One possible explanation of this result is that the influenza vaccination triggers a nonspecific immune response that facilitates protection against adverse COVID-19 events, including death. Additionally, a second plausible mechanism is the induction of an improved cytokine response after the stimulation of immune cells with SARS-CoV-2. Zeng et al. showed similarities in the structure of influenza and coronaviruses and in their binding receptors [8], while Pallikkuth et al. reported a strong relationship of influenza H1N1-specific CD4 T-cell responses with the SARS-CoV-2-specific CD4 T-cell response [32]. Hypothetically, these mechanisms could explain the apparent protective effects of influenza vaccine against COVID-19. However, more studies are necessary to understand the potential role of the correlation between influenza vaccination and COVID-19.

According to our data, the vaccine coverage in patients 62–80 years was the highest; thus, this effect was possibly more pronounced in the elderly population and not significant in patients under 60 years of age. Our data is in line with the recent studies reporting a protective effect of the influenza vaccine against COVID-19-related mortality in the elderly population [13,19,30] and might have relevant implications from a public health perspective. The WHO recommends seasonal influenza vaccinations for priority groups, including pregnant women, children aged between 6 months and 5 years, individuals with chronic medical conditions, healthcare workers and adults aged more than 65 years [33]. The European Centre for Disease Prevention and Control recommends vaccinations for the elderly as a priority [34]. However, a retrospective analysis of the vaccination coverage rates in the general population in 19 EU/EEA Member States in 2016–2017 established that Poland is a country with one of the lowest influenza vaccination coverage rates, and the vaccination coverage rates in older target populations is also one in the lowest in the EU [35]. A study performed by Samel-Kowalik et al. showed that, in November 2020 among adults in Poland, only 5.5% had already been vaccinated against influenza [36], even though adults aged 65 and over are covered by the free vaccination program. Taking into account such an unfavorable vaccination status, an increased risk of COVID-19 infection in the elderly population and a higher chance of severe illness or death due to COVID-19 complications, we therefore highlighted the importance of the influenza vaccine protective effect in Poland and other countries with low influenza vaccine coverage. Given that the influenza vaccine is safe, currently available and, from November 2021, reimbursed for people aged ≥18 years in Poland, it may be an effective and safe option to slow down the coronavirus pandemic and decrease the mortality rate, especially in the elderly. To the best of the authors’ knowledge, this is the first study that has examined the relationship between influenza vaccination and the COVID-19 mortality rate in Poland, as well as in the eastern part of Europe. In addition to the above-mentioned, a major strength of our study is the relatively large cohort size, which includes all individuals registered at the Polish Databank CeZ, a computerized database that is updated regularly.

Although obtaining an influenza vaccination was related to a significant reduction in the risk of death due to COVID-19 infection, this effect might come from the overall health status. People who were vaccinated may be healthier or lead a healthier lifestyle in general and not have preexisting comorbidities in comparison to those who were not vaccinated. However, one could also argue that people with preexisting conditions and poorer health might be more willing to obtain a flu vaccination, because the disease would be much more dangerous to them. Moreover, individuals who agreed to take the influenza vaccination might have a more proactive attitude towards prevention or tend to pay more attention to compliance with the COVID-19 prevention measures, including a respect for social distancing and wearing masks, thus making potential infection less potent; therefore, additional confirmatory studies with randomization are needed.

Our study has several limitations, and the first one to discuss is the presence of misclassification in the vaccination status. We can reasonably assume that it is a case of a nondifferential misclassification, i.e., the incorrect vaccination status assignment has no bearing on COVID-19 mortality. We analyzed the effects of misclassification in terms of sensitivity and specificity, which are the true positives and true negatives, respectively. False positives would involve cases of people who were prescribed a vaccine and filled the prescription but never had the vaccine administered to them. Although possible, the number of vaccines wasted in such a manner should be very low. One reason being the cost, both in time and money, to obtain the vaccine. Another reason is that influenza vaccines are a scarce commodity in Poland and are difficult to come by, especially early in the influenza season. On the other hand, the number of false negatives cannot be reliably estimated based on the data we have.

Secondly, we only analyzed the data from CeZ, and in some regions of Poland, the local government introduced vaccination programs for the elderly and sponsored influenza vaccinations for persons older than 65 years. However, it is impossible to determine in what rate the Polish elderly obtained the free influenza vaccination due to the lack of such data. Therefore, it is likely that our analysis underestimated the number of vaccinated individuals. National vaccine sales statistics [37] indicate that our data comprised about 80% of the total vaccines sold in the country, with a similar age structure of the vaccinated. We can reasonably assume, though, that the older individuals who obtained vaccines through CeZ are similar to those who participated in the local programs, and their mortality due to COVID-19 would not be significantly different. Our simulations indicated that the estimated vaccine effect would only be stronger if false negatives were correctly classified. It is in agreement with an often-quoted statistical observation that misclassification biases results are skewed toward the null.

Thirdly, a major limitation of the study was that the analysis did not allow us to estimate the cause–effect relationship between the vaccine and COVID-19 mortality rate. Our study only focused on COVID-19 mortality in influenza-vaccinated and unvaccinated patients because detailed patient information, particularly regarding the likelihood of developing COVID-19 symptoms, clinical outcomes or hospitalization, was unavailable in the public dataset. In addition, vaccinated patients against influenza differ potentially from unvaccinated patients with respect to health status, health behavior or other unknown biological factors that could affect the differences in clinical presentation and survival. However, we could not address these concerns in our analysis due to the restricted data. More evidence-based studies are needed to further delineate the association between influenza vaccination and COVID-19-related mortality.

## 5. Conclusions

In conclusion, although our findings did not allow us to assume the beneficial impact of the influenza vaccination on the incidence and severity of coronavirus infection, they provided evidence for a correlation between vaccinations against influenza and a reduction in COVID-19 mortality in the elderly population in Poland. In this retrospective study based on electronic health records, we found no evidence suggesting that the influenza vaccine would have a negative impact on populations in terms of COVID-19-related deaths. The administration of the influenza vaccine before the influenza season would reduce the burden of the increased influenza incidence and the risk of an influenza and COVID-19 coinfection and render the essential medical resources able to cope with another wave of COVID-19 hospitalizations.

## Figures and Tables

**Table 1:** Data extracted from CeZ.

Individual	Unique Personal ID Numbers
confirmed COVID-19 infection in the period from 1 January 2020 to 31 March 2021	2,313,496
confirmed COVID-19 infection and death due to COVID-19 infection	93,208
filled the prescription for any influenza vaccine during the 2019–2020 influenza season	1,242,204
confirmed COVID-19 infection in the period from 1 January 2020 to 31 March 2021 and filled the prescription for any influenza vaccine during the 2019–2020 influenza season	116,277

**Table 2:** The mean probability (*p*) of death related to COVID-19 infection among vaccinated and unvaccinated individuals by age group and the odds ratio (OR_u_/OR_v_) between the groups.

Age	Unvaccinated		Vaccinated		Odds Ratio (OR)	
	*p*_u_	95% CI	*p*_v_	95% CI	OR_u/_OR_v_	95% CI
60+	0.128	(0.126; 0.129)	0.121	(0.118; 0.123)	1.18	(1.15; 1.21)
60	0.031	(0.031; 0.033)	0.028	(0.026; 0.029)	1.13	(1.05; 1.23)
65	0.056	(0.056; 0.058)	0.049	(0.047; 0.051)	1.16	(1.09; 1.24)
70	0.094	(0.094; 0.097)	0.080	(0.078; 0.083)	1.19	(1.13; 1.25)
75	0.148	(0.147; 0.152)	0.125	(0.122; 0.129)	1.21	(1.16; 1.27)
80	0.220	(0.218; 0.225)	0.185	(0.180; 0.191)	1.23	(1.18; 1.29)
85	0.308	(0.305; 0.315)	0.261	(0.254; 0.268)	1.26	(1.21; 1.31)
90	0.408	(0.405; 0.418)	0.349	(0.340; 0.359)	1.28	(1.23; 1.34)
95	0.516	(0.511; 0.527)	0.447	(0.345; 0.459)	1.31	(1.26; 1.37)

*p*—probability of death related to COVID-19 infection, u—unvaccinated, v—vaccinated and OR—odds ratio.

## Data Availability

The data are available on request due to restrictions, e.g., privacy or ethical.

## Abbreviations

CeZ	Centrum e-Zdrowia
